# A vision for actionable science in a pandemic

**DOI:** 10.1038/s41467-020-18056-w

**Published:** 2020-09-30

## Abstract

Cornelia Betsch (a psychologist, University of Erfurt), Vittoria Colizza (a computational epidemiologist, INSERM), Sara del Valle (a computational epidemiologist, Los Alamos National Laboratory), Chikwe Ihekweazu (a public health epidemiologist, Nigeria Centre for Disease Control) and Carmela Troncoso (a data security specialist, EPFL) talked to Nature Communications about their experience with COVID-19 response and their vision on a new system for disease surveillance and control, providing a view on how this should interact with policy making.

**Figure:**
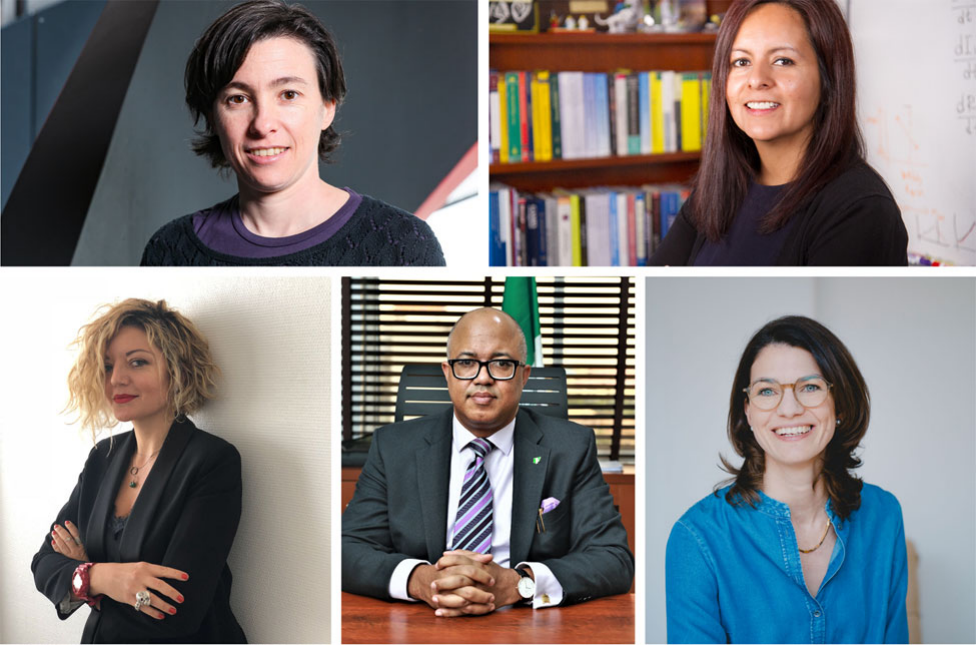
(clockwise from upper left): Carmela Troncoso, Sara del Valle, Cornelia Betsch, Chikwe Ihekweazu, Vittoria Colizza

How were you involved with COVID-19 response?

**C.B**.: When there were 17 confirmed COVID-19 cases in Germany in late February, we started preparing a weekly COVID-19 Snapshot Monitoring (COSMO)^1^. COSMO went online one week later when we had 159 cases. Reducing transmissions of COVID-19 requires large-scale behavioural change. Knowledge about the determinants of behaviour is a crucial lever to instigate and maintain behavioural change^2^. Thus, we assessed people’s risk perceptions, trust in government and science, knowledge, acceptance of measures, psychological strain and mental health aspects of the outbreak. Currently, we have surveyed almost 16,000 participants, approximately 1000 per week. The results are published on a website (www.corona-monitor.de^3^) and the government and other relevant stakeholders receive additional policy briefs. We cooperated with the World Health Organization’s Regional Office for Europe who adapted the study protocol^4^, and approximately 40 countries intended to employ similar study protocols to deliver evidence about the psychological aspects of this crisis. In Germany, this assessment allowed identifying groups with special needs, such as families with children under 14 years of age, and provided feedback about the uptake of newly recommended behaviours, such as mask wearing.

**V.C.:** I co-lead the modelling activities of the French Task Force REACTing (Research and action targeting emerging infectious diseases), a multi-disciplinary collaborative network of researchers created few years ago to prepare and respond against emerging infectious disease epidemics. We mobilized as soon as mid-January in collaboration with Sante publique France (French national authority for public health) for COVID-19 response. Our role has been to identify the needs of authorities and inform Sante publique France and the Ministry of Health all along the successive phases of the epidemic. During the early phase, we provided estimations for the risk of importation in France and in Europe, suggesting revisions of the case definition for suspect cases as control measures in China changed and outbreak reached other countries. With importations detected in several areas of the world, we alerted authorities that an estimated large portion of imported cases (60%) was not ascertained, suggesting that silently spreading epidemics were possibly already established in the territory. Then, the big part of our work focused on the evaluation of the lockdown in France and proposal of exit strategies. We keep monitoring the epidemic activity and provide operational indicators to anticipate its evolution in the next few weeks and prepare for the Fall.

**S.D.V**.: I’m still involved in several COVID-19-related efforts in support of the Center for Disease Control and Prevention (CDC), the Federal Emergency Management Agency (FEMA), the Department of Energy (DOE), Los Alamos National Laboratory (LANL), and the State of New Mexico. Our team supports these local and federal organizations through epidemic modelling by providing science-based decision support regarding the impact of mitigation strategies and forecasts. For example, our team has been releasing U.S. (state-level) and global (country-level) forecasts twice a week since the beginning of April (https://covid-19.bsvgateway.org).

**C.I**.: I lead the Nigeria Centre for Disease Control (NCDC), Nigeria’s national public health institute. Our agency is leading the public health response to COVID-19 in Nigeria. The role of NCDC includes providing scientific guidance, coordination and leadership of public health response activities—surveillance and epidemiology, laboratory services, risk communications, logistics and supply chain. At the global level, I serve on advisory committees to the leadership of Africa CDC and WHO. **C.T**.: During this pandemic I have been part of the DP3T (Decentralized Privacy-Preserving Proximity Tracing) group, a collective of scientists working on a privacy-preserving proximity tracing app to help with COVID-19 containment. We have given support to the Swiss government in the design and implementation of their app SwissCovid.

How has epidemiological data collection and sharing worked in this pandemic and do you think there are grounds for implementing a real global platform? 

**V.C.:** This is the first pandemic (or recent major epidemic) where we witnessed an unprecedented sharing and large-scale availability of epidemic data. Few days into the outbreak and a first dashboard was created. Technological factors, widespread interest for tracking phenomena, pandemic context of concern, scientific community at large focusing on the pandemic, and open data movement likely converged to make this sharing possible, compared to previous emergencies. Over time, and given the dimension of concern affecting every citizen, also governments felt the pressure for making data available, creating interactive dashboards and filling GitHub repositories.

Large-scale access promoted scientific progress into our understanding of the epidemic, allowing a multiplicity of perspectives and approaches from different groups in the world at levels unseen before. And it also boosted scientific dissemination and large-scale communication to the general population (directly, and through the media) as an important side-effect.

Still, we are a long way from sharing data with information at the granularity level needed for in-depth studies. Epidemic data collected throughout an ongoing outbreak suffer from multiple sources of bias (missing data, delays, underestimation) and would require additional information than generally provided to assess and correct them. Common standards are also lacking (COVID-19 deaths classification or case definition, for example), or may evolve over time with increased knowledge or following changing policies, country by country. Finally, breakdowns of the data providing additional stratifications (by age, risk factor, setting, etc.) hit the limits imposed by privacy concerns.

The main obstacle to a global perspective for data collection is represented by the clash between the national dimension of public health (in terms of policies, standards, privacy regulations, epidemic management, classifications, resources, capacity, sovereignty) and the global needs and challenges posed by pandemics.

COVID-19 pandemic led global economic uncertainty to a record high. This crisis is probably also the best opportunity we have to address this challenge and radically improve our response to future crises.

**S.D.V**.: Although several epidemiological data streams such as cases, deaths, tests, and genetic sequences have become available at unprecedented speeds at the state and, in some cases, regional level (e.g., county, municipality), there are more data sources that would be beneficial for epidemic modellers. Unfortunately, real-time or daily information on hospitalizations, ICU usage, ventilator usage, testing, and patient-level information (i.e., line lists) at the regional level (and even state level) are sparsely available. These types of data can increase the accuracy of epidemic models given the heterogeneity in COVID-19 spread.

I think this pandemic has highlighted the need for a global disease forecasting centre or initiative whose goals would include: (1) data collection, (2) monitoring/tracking, (3) understanding, (4) forecasting, and (5) analysing the impact of different intervention strategies in order to provide decision support during emerging and re-emerging epidemics and pandemics.

In general, I’m optimistic about the potential for future data sharing agreements, not just when we’re facing a pandemic, but always.

**C.I**.: In Nigeria, getting our epidemiological data collection and sharing capacity right was one of the biggest priorities at the beginning of the pandemic. One critical step was the deployment of SORMAS (Surveillance Outbreak Response Management and Analysis System)—a real-time web-based software for outbreak and epidemic surveillance. SORMAS was developed and first used in Nigeria in 2014 to support the Ebola response. Since then, we have adapted its use for other epidemic prone diseases in Nigeria and for COVID-19. The use of SORMAS has strengthened Nigeria’s capacity to collect, analyze data and use this for critical decision making. This has worked to a very large extent for us, as the states in Nigeria share their COVID-19 data through SORMAS with uniform indicators to enable decision making.

Since the first case in Nigeria, we have published our data through a daily situation report. Our policies have also been largely driven by data provided. In the region, this data is provided regularly to the West African Health Organisation and Africa Centres for Disease Control. Every week, we receive a detailed situation from both organisations, on the situation in West Africa and the African continent respectively. The knowledge of the situation in other regions is an important resource for us, especially as we begin discussions around border opening. The global platform provided by WHO, through its regular situation reports and guidelines, has also supported our work as we learn from other countries.

However, there are grounds for the global community to strengthen the current structure. Countries must see data sharing as a responsibility and obligation for global good. This means sharing timely data in an open manner, with minimal political interference. The countries with the resources to lead on this have unfortunately not done so.

What are the obstacles now, apart from the initial scarcity of data, to a more thorough validation of epidemiological models to demonstrate their robustness (e.g. showing predictive power outside their spatial and temporal fitting range)?

**V.C.:** The biggest challenge is undoubtedly human behaviour. Preventing transmission of respiratory infections means altering routine behaviours of our everyday life and habits deeply rooted in our cultures. Models can integrate human behaviour in normal peacetime conditions (e.g. number of contacts in different settings, daily mobility to school or work, travel, etc.), and to a good extent under certain imposed restrictions (school closure, travel restrictions). But they may fail to capture adaptive behaviours of the population in response to the evolving epidemic situation and/or control policies. How many people will stop kissing and hugging to avoid risk of transmission? How many will comply or will be able to comply to stay-at-home messages given their professional, socioeconomic and family situation? What fraction of parents will send their children to school when they reopen? How many people will wear a mask? Will adherence to preventive measures fade with time?

There is a non-trivial interplay of factors shaping, directly or indirectly, an outbreak dynamics—transmission, behaviours, culture, perception of risk, control measures—thus making the human component central to epidemic modelling. 2009 H1N1 influenza pandemic showed a glimpse of this with the vaccination campaigns. By Fall, risk was perceived to be very low, resulting in unexpectedly low vaccination uptakes. But in these first few months, COVID-19 pandemic exacerbated this interplay to new levels. Risk perception varied wildly over time, authorities adopted unprecedented measures for control, individuals largely showed compliance to policy and recommendations, however with notable exceptions (in France, widespread opposition to reopening schools after lockdown, low adoption of the app for digital contact tracing). Moreover, prior studies and current evidence clearly show that these aspects are strongly dependent on culture and prior experiences.

Lacking prior historical examples for model parameterization (because not as severe as COVID-19, or not occurring in modern conditions), some of these behaviours can nowadays be measured in almost real time to inform models. This pandemic saw the wide diffusion of mobility tracking projects for the automated collection and analysis of changes in mobility, based on collaborations of academic teams with telcos, or provided by tech giants granting access to their own mobility stats. Similarly, perceptions and attitudes can be monitored with social media analyses, which proved its efficacy in recent epidemics. Other data need instead more traditional surveys to quantify certain behaviours, for example social contact mixing during and after lockdown, adoption of physical distancing, mask and hygienic measures, health-seeking behaviour for screening, and others. These investigations were rapidly performed thanks to already available protocols, rapid ethics approval processes due to the emergency, or exploiting already existing online participatory platforms (e.g. COVIDnet.fr adapted from the surveillance of influenza-like-illness in the general population, part of the Influenzanet network at European level).

With these data, models can be accurately parameterized up to nearly the present. Then, short-term projections assume plausible conditions for these behaviours in the following weeks (e.g. the same tendency in adoption of preventive behaviours reported in the last period), whereas scenario analyses can provide longer term indications of how the epidemic trajectory could evolve under a variety of conditions (e.g. reopening of schools, different mitigation policies, etc.). Robustness of model predictions will therefore depend crucially on our ability to describe human behaviours over time. Models, however, will not be able to capture unexpected events, such as for example the exodus registered prior to the implementation of lockdown (from Paris towards the coastal regions in France or from the North to the South of Italy).

**S.D.V.:** Aside from data scarcity, we need ensembles of various modelling approaches (similar to hurricane forecasting) to make sure the approaches and forecasts are robust. If you’re looking at one model only, you’re not seeing the full picture. We need to be able to start testing the limits of these models by actually trying to forecast different diseases across different regions and address limitations.

Decision makers ask for “actionable” models, i.e. specific policies can be implemented directly and reliably into their mechanisms and/or parameters, with reasonably fast turnaround times, but more complex models reasonably ask for more complex data. How far are we in terms of routine data collection (including socio-demographic and behavioral data)? On the other hand, can models implement policies reliably and what policy directions have models provided in this pandemic?

**C.B**.: The dynamic COVID-19 situation required fast action. For example, German schools were closed only ten days after the first person died from COVID-19. Thus, data that could inform the communication around the lockdown and provide feedback to people in charge were immediately necessary, e.g., whether people understood the risks associated with COVID-19, whether they accepted the lockdown, or which groups had special needs. No routine data collection mechanism existed that could be instantly used. Interestingly, a recent paper analysed the most frequent terms in more than 5,700 papers that had already been published on COVID-19^5^, and none of the frequent topics referred to behaviour or similar concepts. The maybe closest term was “knowledge”—however, it is important to note that even though knowledge is important, more knowledge will not necessarily change behaviour^6^. While data collection efforts are now underway or still developing, a comparison of the volume of research with the medical and related fields shows that only few studies that address behavioural change have been published (but meanwhile there is a high number of preprints). To enhance pandemic preparedness, a careful post-evaluation of cross-country data will be necessary. Surveys such as the “Behavioural Insights Tool” 4 provide the extraordinary chance to compare data globally. The tool needs to be evaluated and revised to ensure readiness in subsequent phases of this pandemic and a potential future pandemic. We should further use the data to build new models that link psychological survey data, epidemiological data of the disease dynamics^7^, (social) media data, and policy data to understand the acceptance of measures, their impact on the epidemic, and the mediating and moderating role of psychological and cultural factors^8^. There is a great need to improve existing models and integrate data from different fields.

**V.C.:** Complex models ask for complex data and… a lot of time! Science for outbreak response is very different from science in peacetime. Modelling research typically needs to strike a balance between question to answer on one side vs. available data, reasonable amount of detail, and suitable approach on the other. Science for outbreak response brings two additional challenges: data is poor, time is extremely limited. So, data-hungry models are generally not well suited for the initial phase of an emerging disease epidemic, as we do not have enough knowledge to parameterize them. Or, they may heavily rely on data measured in peacetime conditions that are heavily disrupted during the epidemic (e.g. contacts and mobility during lockdown to curb COVID-19), opening the door to many untested assumptions.

The role of models in guiding policies was most evident in the implementation of the lockdown to curb the first wave in Europe. With a collection effort for behavioural data at its start, at a moment where knowledge was still extremely limited and based exclusively on the outbreak in China, models were able to predict the saturation of the healthcare system in the most affected countries unless drastic measures were implemented. Independent studies based on different approaches were reaching the same conclusions, providing a much-needed validation in such uncertainty. Subsequently, models evaluated the impact of lockdown, estimated the duration required to suppress the epidemic activity, and proposed progressive strategies to phase out lockdown. Even in 100% uncertainty on the role of transmissibility of children, models were able to identify protocols for school reopening that would maintain the epidemic under control, showing how models can be used to narrow down uncertainty.

**S.D.V.:** Models are intended to assess different intervention strategies and provide insights so policymakers can balance the pros and cons of possible scenarios. We have pretty reliable information regarding socio-demographic factors through census data. However, behavioural data is very hard to come by. Although mobile data is currently being used to measure behaviour (i.e., mobility), it only captures one aspect of the problem. We still don’t have information about facemask usage, hygiene compliance, and other social distancing measures taken by age, gender, and other socio-demographic factors. Given the limitations in data availability, models can assess “what if” scenarios (i.e., what if 50% of the population wears a facemask) to provide insights, but these assumptions are hard to validate with current data.

During COVID-19, models have been useful in several ways including:

- Identifying “hot spots” and overall trends through modelling and forecasting approaches
- Identifying future hospitalization, ICU, and ventilator usage (through forecasting approaches)
- Assessing the impact of non-pharmaceutical mitigation strategies in reducing spread (e.g., facemask, social distancing, quarantining, isolation)
- Assessing the impact of school and workplace reopening scenarios

**C.I**.: Firstly, we must be wary of adapting a “one size fits all” approach for countries. Countries are at varying aspects of their response and have different response capacities. In a pandemic and during outbreaks generally, reducing the risk of transmission and fatality rate should be priority. Public health responders should not be distracted from their primary response by focusing only on collecting complex data. The collection of complex data can be done alongside public health response but in a situation of limited resources, the public health response must be prioritised.

In Nigeria, we have made progress with the collection of routine public health data. However, collecting public health data is largely dependent on demographic data managed by non-public health agencies. Examples of this are population size, number of children in school or out of school etc. In the absence of recent data, we have to work with estimates which reduce the level of accuracy of models.

Models can be used to guide and implement policies, but we must be aware of the limitations and constraints of mathematical models. As we are responding to a novel virus, unexpected events are a repeated feature. Models cannot anticipate some of these events, and it is important to be aware of this.

In responding to COVID-19, models have guided our introduction and review of non-pharmaceutical interventions. This includes the closure of schools, identification of hotspots, guidance on inter-state travels etc. However, these decisions have been based on models and other indicators such as data from routine surveillance activities.

What are the main concerns in a more widespread and deeper public surveillance in terms of privacy and how can these concerns be addressed without hindering disease prevention and control?

**C.T**.: Wide surveillance has the potential to undermine our freedom and as such to completely change society. Democracy requires freedom for people to make decisions on their own. For people to be free it is important that they don’t feel observed and they cannot be manipulated. Large data collection, and the capabilities that these data can provide, results in an asymmetry of power: those with data about particular persons or groups gain great power over those people: they can know what they do, what they like, what they think… and use this information to influence their actions or take decisions to control or limit them. Democracy cannot exist without data privacy.

Do you think these have been tackled satisfactorily, for instance in the first contact tracing apps that have been released/are to be released in the near future?

**C.T**.: The privacy-by-design apps that are currently being deployed can perform the task necessary for containment (notifying potentially exposed people) without requiring the collection of vast amounts of data. Therefore, they do not require trust on the institutions that run the system, and prevent abuses of data by design.

These apps are a clear example that surveillance is not necessary to achieve protection. I believe that with more collaboration between epidemiologists and professionals of the medical domain, and technologists, we will be able to develop many more solutions for disease prevention and control that are respectful of our cherished societal values.

There have been several examples of how scientific indications, in general, have been used to support political decisions. From your perspective, how do you expect a decision maker to use model outputs?

**C.B**.: Scientific evidence is often conflicting and sometimes create debates in science and even the public. It is important that the existence of scientific uncertainty and public debates does not lead to the false conclusion that science can be disregarded. Politicians face the additional challenge of ensuring that their decisions reflect a broad spectrum of societal values and needs, for which the feedback mechanism is gaining popularity and being re-elected. For example, it may make sense to prevent transmissions especially in densely populated areas^9^. However, will a politician decide to quarantine people only in some areas, or provide a vaccine first in a certain area and require other areas to wait for a longer time? Scientific advisory boards can help establish trusted relationships and provide governments with valuable input. Research units could be installed in governments that help to substantiate policies with existing and new data. Various techniques exist to make scientific expertise actionable, e.g., expert elicitation^10^, which systematically assesses experts’ subjective probabilities when existing knowledge is insufficient; or the “college of owls”^11^, where scientists judge the level of scientific consensus regarding a pertinent question (e.g., regarding the impact of prioritising densely populated areas for earlier access to vaccination). The latter assessment determines the need for political justification—the less consensus, the greater is the amount of justification that is required.

**V.C.:** Communication of scientific findings to health authorities and decision makers is probably the most difficult task in outbreak response, and—for me—also one that carries huge responsibilities. I believe the role of science remains in the presentation of achieved evidence, specifically highlighting uncertainties, underlying assumptions, and—most importantly—limitations. Evidence then needs to be evaluated considering a much broader perspective, going well beyond protecting people’s health and factoring in other critical aspects such as economy, resources, capacity, sustainability, wellbeing. Science support to policy works best if it is performed routinely also in peacetime, e.g. to inform on vaccination policies, recommendations, improvement of surveillance schemes. It strengthens the interactions for when an emergency hits.

**S.D.V**.: Projection modelling results are intended to assess different “what if” scenarios and help decision makers weigh the pros/cons of potential decisions and outcomes. Forecasts are intended to provide information pointing to hot spots and hot moments to be better prepared in terms of resource allocations.

Much of the uncertainty in both modelling and decision making comes from the fact that, for communicable diseases especially, we are dealing with human behaviour. During this pandemic we have also experienced some of the most drastic measures in recent history, that have depended/depend on human behavior. What information have we gathered in terms of compliance to, say, limitations to movement and/or usage of protective masks/other personal sanitation?

**C.B**.: The weekly data that were collected in Germany, for example, gave insights into how people acquired new information: in the first week, during which the media reported on the loss of taste and smell as a symptom of COVID-19, only 24% of the respondents knew this symptom; in the following week, 68% were aware of it (corona-monitor.de). Thus, most people closely followed the news that delivered important information; in Germany, especially the public media gained importance. The data also provided insight into how much people complied with single measures or rejected measures in general. In March and April 2020, for example, the degree to which measures were considered exaggerated correlated highly with mobility data from mobile phones: When data indicated high compliance, mobility was very low; when the proportion of people who considered the measures exaggerated increased, mobility increased to the same extent. For specific measures, such as mask wearing, the data provided evidence of the effect of policies and the foundations for communication strategies. Similar to many other countries, at the beginning of the pandemic, mask wearing was not recommended due to the shortage of medical N95 masks. When it became evident that even fabric masks could prevent transmission and protect other people, mask wearing in stores and public transport became mandatory in Germany. The data show a drastic increase due to the policy shift: 34% vs. 77% of people were reported wearing masks the week before the policy was implemented and the week after the policy came into effect, respectively. The data also showed that the policy change was also (but somewhat less) successful in reaching approximately a fifth of the population who believed that the measures were exaggerated^3^. To a limited extent, the data could also motivate political action that indirectly addresses the opposition to the measures—e.g. by addressing correlates of opposition, such as economic worries, or by developing support mechanisms addressing population subgroups that have been identified as vulnerable. However, it is important to note that any obtained correlation should not be interpreted as causal; interventions should therefore be substantiated with existing behavioural change knowledge^8^

**V.C.:** Compliance generally varies by country, implemented measures, epidemic context experienced, and it is affected by education, socio-economic level, and risk perception. During the first COVID-19 pandemic wave, several mobility tracking projects in Europe reported reductions in displacements around 60–70% due to lockdown. Reductions were, however, non-uniform across regions, due to rigid constraints (professional sectors mostly impacted by the lockdown), demographic and socio-economic indicators (larger percentage of working age class, higher propensity to telework for higher socio-economic levels).

In France, we also found an association with a risk aversion component, leading to a higher mobility reduction in those regions that were mostly affected by the epidemic. Systematic compliance to preventive measures (hygienic measures, physical distancing, use of mask) is generally decreasing. Kisses and hugs were avoided by about 90% of the population during lockdown, and saw a linearly decreasing trend since lockdown lifting, reducing compliance to 70% of the population in the first week of July. The use of masks has been steadily increasing from late March (10%) to late May (50%), to stabilize around that value since.

Clearly these estimates may rapidly vary following policy changes (as the newly imposed use of masks in closed settings) or sudden resurgence of cases in specific region altering the perception of risk. It will be interesting to explore in the future if our habits concerning hygienic measures have been altered by COVID-19 pandemic, as it was reported for South-Asian countries following SARS epidemic.

**S.D.V**.: Many organizations are collecting mobility patterns based on cell phone data, which can allow modellers to measure how changes in mobility are contributing to disease spread. Similarly, researchers are using social media data such as Twitter to estimate sentiment regarding facemasks and hygiene. However, one of the limitations of Twitter data is the lack geospatial information (i.e., <5% of tweets are geotagged), therefore, it’s difficult to develop robust spatial analysis based on social media data. Nevertheless, we have more ways now to measure different types of behaviour and our hope is that new databases focused on behavioural data will become available in the near future to better assess and measure human behaviour in response to epidemics and pandemics.

How has policy communication worked during the pandemic and is there evidence (now but also in previous experience) that correct communication practices can influence public compliance?

**C.B**.: Only a few evaluations of communication strategies have been published so far. A first post-hoc evaluation of China’s handling of the crisis has been published, which criticises that the “Wuhan government did not infuse a scientific risk communication into policy decision, (…) stalled reporting and handled the information publicity in an ambiguous way (…) which worsened the circulation of rumours and led to public panic to some extent”^12^. The Norwegian strategy has been evaluated as very successful due to a “collaborative and pragmatic decision‐making style, successful communication with the public, a lot of resources, and a high level of citizen trust in government”^13^. Both observations might be relevant to other countries as well. In-crisis evaluation of the effectiveness of communication strategies is inherently difficult due to a lack of control groups. Therefore, behavioural experiments that assess causal effects of communication measures have great value. Often, studies show mixed evidence, for example, on communicating uncertainty^14^. With a sharp increase in COVID-19 cases, behavioural scientists made research rapidly available on preprint servers, which suggested additional valuable “how-tos” to the stakeholders (e.g., that emotional, empathy-related communication can increase mask wearing or physical distancing^15^). This active and self-confident role of behavioural scientists^16^ also fuelled the debate about the general validity, replicability and policy-readiness of socio-behavioural research^17^. During the research process, preregistrations and registered reports may increase trustworthiness of findings. When it comes to synthesizing evidence, it has been recommended to combine existing evidence from controlled research settings, preferably summarised in meta-analyses, with ad-hoc evidence collected during the pandemic^8^. Longitudinal or serial cross-sectional studies can assess indicators for successful crisis communication, such as trust. For example, German data show that trust in the government was highest when cases peaked, and positively and continuously related to the acceptance of and compliance with measures^3^. Regular data collection during the crisis can serve as an early warning system, e.g., by indicating decreasing trust, and serve as evidence base to develop and pre-test communication strategies.

**C.I**.: In Nigeria, the introduction and review of policies has been communicated using a top-bottom approach. At the beginning of the COVID-19 outbreak, the President of Nigeria held briefings to announce the introduction of policies such as lockdowns. In addition, the Presidential Task Force (PTF) on COVID-19 has provided regular updates based on the review of policies. The PTF brings together a diverse group of stakeholders from the health sector and other sectors such as education, transportation, information etc, to allow for a wide range of consultation and harmonisation of policies.

In addition to the top-bottom approach, each sector is responsible for developing strategies to communicate with relevant stakeholders. For example, the Ministry of Education has hosted virtual town hall meetings with school owners, parents and student representatives to address school closure policies. The PTF has also engaged with community leaders such as traditional rulers, religious leaders, professional associations and other defined groups to support communication to members of the public and by so doing, build trust in the public health response.

The public compliance to COVID-19 policies in Nigeria has relied strongly on strong public communication. By engaging with stakeholders such as business owners, traditional and religious leaders, we recorded a high level of compliance to closure of large gatherings, schools, churches etc. However, compliance is also largely dependent on personal responsibility. Our COVID-19 communications campaign tagged “Take Responsibility” is a reminder that stopping the outbreak relies on people taking responsibility to not host gatherings, maintain physical distance, avoid non-essential movement and other measures.

*The interview was done by Nature Communications editors Lorenzo Righetto, Brittany Cardwell, Catherine Smith, Sonja Schmid and Iryna Omelchenko*.

## References

[1] Betsch, C. et al. Germany COVID-19 Snapshot MOnitoring (COSMO Germany): monitoring knowledge, risk perceptions, preventive behaviours, and public trust in the current coronavirus outbreak in Germany. 10.23668/PSYCHARCHIVES.2776 (2020).

[2] Betsch, C. How behavioural science data helps mitigate the COVID-19 crisis. *Nat. Human Behav.*10.1038/s41562-020-0866-1 (2020). 10.1038/s41562-020-0866-1 PMC7101898 32221514

[3] COSMO group. Corona-Monitor. www.corona-monitor.de. (2020)

[4] WHO Regional Office For Europe. COVID-19 Snapshot MOnitoring (COSMO Standard): monitoring knowledge, risk perceptions, preventive behaviours, and public trust in the current coronavirus outbreak—WHO standard protocol. 10.23668/PSYCHARCHIVES.2782 (2020).

[5] Tran BX (2020). Studies of novel coronavirus disease 19 (Covid-19) pandemic: a global analysis of literature. Int. J. Environ. Res. Public Health.

[6] Simis MJ, Madden H, Cacciatore MA, Yeo SK (2016). The lure of rationality: why does the deficit model persist in science communication?. Public Underst. Sci..

[7] Maier BF, Brockmann D (2020). Effective containment explains subexponential growth in recent confirmed COVID-19 cases in China. Science.

[8] Habersaat, K. B. et al. Ten considerations for effectively managing the COVID-19 transition. *Nat. Human Behav.*10.1038/s41562-020-0906-x (2020) 10.1038/s41562-020-0906-x 32581299

[9] Arita I, Wickett J, Fenner F (1986). Impact of population density on immunization programmes. J. Hyg..

[10] Morgan MG (2014). Use (and abuse) of expert elicitation in support of decision making for public policy. Proc. Natl Acad. Sci. USA.

[11] Collins, H. & Evans, R. *Why Democracies Need Science*. (John Wiley & Sons, 2017).

[12] Zhang L, Li H, Chen K (2020). Effective risk communication for public health emergency: reflection on the COVID-19 (2019-nCoV) outbreak in Wuhan, China. Healthcare.

[13] Christensen, T. & Lægreid, P. Balancing governance capacity and legitimacy: how the Norwegian government handled the COVID‐19 crisis as a high performer. *Public Adm. Rev.*10.1111/puar.13241 (2020). 10.1111/puar.13241 PMC7280699 32836445

[14] van der Bles AM (2019). Communicating uncertainty about facts, numbers and science. R. Soc. Open Sci..

[15] Pfattheicher, S., Nockur, L., Böhm, R., Sassenrath, C. & Petersen, M. B. The emotional path to action: empathy promotes physical distancing and wearing face masks during the COVID-19 pandemic. https://osf.io/y2cg5 (2020). 10.1177/0956797620964422 32993455

[16] Bavel, J. J. V. et al. Using social and behavioural science to support COVID-19 pandemic response. *Nat. Human Behav.*10.1038/s41562-020-0884-z (2020). 10.1038/s41562-020-0884-z 32355299

[17] Ioannidis JPA (2005). Why most published research findings are false. PLoS Med..

